# High-Throughput Detection of Autoantigen-Specific B Cells Among Distinct Functional Subsets in Autoimmune Donors

**DOI:** 10.3389/fimmu.2021.685718

**Published:** 2021-05-24

**Authors:** Bryan A. Joosse, James H. Jackson, Alberto Cisneros, Austin B. Santhin, Scott A. Smith, Daniel J. Moore, Leslie J. Crofford, Erin M. Wilfong, Rachel H. Bonami

**Affiliations:** ^1^ Department of Medicine, Division of Rheumatology and Immunology, Vanderbilt University Medical Center, Nashville, TN, United States; ^2^ Department of Biomedical Sciences, School of Medicine Greenville, University of South Carolina, Greenville, SC, United States; ^3^ Department of Medicine, Division of Infectious Diseases, Vanderbilt University Medical Center, Nashville, TN, United States; ^4^ Department of Pathology, Microbiology and Immunology, Vanderbilt University Medical Center, Nashville, TN, United States; ^5^ Vanderbilt Institute for Infection, Immunology, and Inflammation (VI4), Nashville, TN, United States; ^6^ Department of Pediatrics, Division of Endocrinology & Diabetes, Vanderbilt University Medical Center, Nashville, TN, United States; ^7^ Department of Medicine, Allergy, Pulmonary, and Critical Care, Vanderbilt University Medical Center, Nashville, TN, United States

**Keywords:** B cells, B cell receptor (BCR), autoimmune disease, autoantigen, myositis, Sjogren’s syndrome, systemic sclerosis (scleroderma), type 1 diabetes

## Abstract

Antigen-specific B cells (ASBCs) can drive autoimmune disease by presenting autoantigen to cognate T cells to drive their activation, proliferation, and effector cell differentiation and/or by differentiating into autoantibody-secreting cells. Autoantibodies are frequently used to predict risk and diagnose several autoimmune diseases. ASBCs can drive type 1 diabetes even when immune tolerance mechanisms block their differentiation into antibody-secreting cells. Furthermore, anti-histidyl tRNA synthetase syndrome patients have expanded IgM^+^ Jo-1-binding B cells, which clinically diagnostic IgG Jo-1 autoantibodies may not fully reflect. Given the potential disconnect between the pathologic function of ASBCs and autoantibody secretion, direct study of ASBCs is a necessary step towards developing better therapies for autoimmune diseases, which often have no available cure. We therefore developed a high-throughput screening pipeline to 1) phenotypically identify specific B cell subsets, 2) expand them *in vitro*, 3) drive them to secrete BCRs as antibody, and 4) identify wells enriched for ASBCs through ELISA detection of antibody. We tested the capacity of several B cell subset(s) to differentiate into antibody-secreting cells following this robust stimulation. IgM^+^ and/or IgD^+^, CD27^-^ memory, memory, switched memory, and B_ND_ B cells secreted B cell receptor (BCR) as antibody following *in vitro* stimulation, whereas few plasmablasts responded. Bimodal responses were observed across autoimmune donors for IgM^+^ CD21^lo^ and IgM^-^ CD21^lo^ B cells, consistent with documented heterogeneity within the CD21^lo^ subset. Using this approach, we detected insulin-binding B cell bias towards CD27^-^ memory and CD27^+^ memory subsets in pre-symptomatic type 1 diabetes donors. We took advantage of routine detection of Jo-1-binding B cells in Jo-1+ anti-histidyl tRNA synthetase syndrome patients to show that Jo-1-binding B cells and total B cells expanded 20-30-fold using this culture system. Overall, these studies highlight technology that is amenable to small numbers of cryopreserved peripheral blood mononuclear cells that enables interrogation of phenotypic and repertoire attributes of ASBCs derived from autoimmune patients.

## Introduction

B lymphocytes contribute to immune responses by presenting antigens to T cells, secreting cytokines, and differentiating into antibody-secreting cells. Autoantibodies are frequently used to predict and diagnose autoimmune diseases, highlighting the important role that B cells play in promoting autoimmunity (1–7). In some diseases, such as Sjögren’s syndrome, systemic lupus erythematosus, and rheumatoid arthritis, autoantibodies have pathologic function *via* immune complex formation (8). In others, such as type 1 diabetes, autoantibodies are not directly pathogenic (9); rather, it is the antigen-presenting function of the B cell that is essential for disease (9–13). Autoimmune disease treatments such as prednisone, rituximab, or abatacept involve broad immune suppression. For example, rituximab globally depletes B cells which is effective at treating several autoimmune diseases, including rheumatoid arthritis, systemic lupus erythematosus, anti-histidyl tRNA synthetase syndrome, and systemic sclerosis (14–20). Rituximab is well-tolerated in adults, but results in diminution of vaccine responses, a key consideration for treatment of pediatric autoimmune diseases such as type 1 diabetes (21). Therapies that selectively target ASBCs would avoid the problem of broad immune suppression and should thus be safer. Selection elimination of anti-insulin B cells prevents disease in type 1 diabetes-prone mice (22); targeting ASBCs may thus offer an effective alternative to broad immunosuppression for autoimmune disease prevention and treatment. Understanding the mechanisms that govern immune tolerance breach by autoreactive B cells requires identification and study of ASBCs.

B lymphocytes express antigen-specific, membrane-bound B cell receptors but are not a major source of circulating antibody. Rather, B lymphocytes must receive the right stimulation to differentiate into plasmablasts or plasma cells that secrete BCR as circulating antibody (23). Different immune checkpoints govern whether autoreactive B cells 1) expand, 2) undergo mutation and affinity maturation, and 3) differentiate into antibody-secreting cells (23, 24). In Sjögren’s syndrome, sustained Ro60 autoantibody production is due to continual generation of plasmablasts from ASBCs, rather than long-lived plasma cells, suggesting continual autoreactive B cell seeding of the peripheral repertoire is required (25). Studies in mice show that autoantigen-specific B cells (ASBCs) can retain disease-relevant autoantigen-presenting function even when immune tolerance mechanisms block their differentiation into autoantibody-secreting cells (26–28). This points to a need to identify the specific mechanisms by which ASBCs escape immune tolerance to expand and drive pathology, a process which may differ between autoimmune diseases.

Methods have been developed to track ASBCs in the broad repertoire that are as rare as 1 in 20 million cells (29). Many different B cell subsets can potentially contribute to a protective or autoimmune response that may have different responsiveness to specific stimuli. For example, whereas naïve B cells proliferate in response to BCR stimulation, anergic (B_ND_) and CD21^lo^ B cells do not (30, 31). B_ND_ and CD21^lo^ subsets may serve as reservoirs for autoreactive B cells in several autoimmune diseases, including type 1 diabetes, Sjögren’s syndrome, anti-histidyl tRNA synthetase syndrome, and systemic sclerosis (32–36). We sought to develop high-throughput stimulation and screening methods to identify ASBCs among total PBMCs using ELISA detection of BCRs secreted as antibody. Given the signaling differences present among autoreactive-prone B cell subsets of interest, we examined the potential of each of these subsets to respond to robust stimulation *in vitro* that includes BCR, CpG, CD40L, IL-21, and BAFF stimulation (29). We further tested the capacity of several memory B cell subsets and plasmablasts to differentiate/secrete BCR as antibody in this culture system as a mean to identify ASBCs within a polyclonal repertoire.

## Materials and Methods

### Participant Selection and Clinical Information

Systemic sclerosis and Jo-1+ anti-histidyl tRNA synthetase syndrome patients were selected from enrollees in the Vanderbilt Myositis and Scleroderma Treatment and Investigative Center (MYSTIC) cohort. A subset of systemic sclerosis patients was defined as having interstitial lung disease if a radiologist determined that fibrosis was present on a CT scan. Sjögren’s syndrome patients were selected from enrollees in the B Lymphocytes in Sjögren’s syndrome (BLISS) at Vanderbilt University Medical Center. Patients were eligible for the study if they were 18 years or older and had been diagnosed with systemic sclerosis by a rheumatologist or pulmonologist or Sjögren’s syndrome by a rheumatologist. Clinical data were collected from the electronic medical record. One donor was obtained from the Human Immunology Discovery Initiative (HIDI) cohort. Additional autoimmune diagnoses were obtained *via* patient-reported questionnaire. These studies were approved by the Vanderbilt Institutional Review Board. Participant characteristics are outlined in **Table 1**.

**Table 1:** Demographics for participants included from MYSTIC, BLISS, and HIDI cohorts.

SubjectID	Cohort^a^	Gender	Age Range (years)^b^	Immunomodulatory agents at enrollment^c^	Autoimmune disease diagnosis^d^	Autoantibodies^e^
1	MYSTIC	F		MMF	SSc/ILD	Scl70
2	MYSTIC	F		None	SSc/ILD	Scl70
3	MYSTIC	M		None	SSc/ILD	Scl70
4	MYSTIC	F		None	SSc/ILD	Scl70
5	MYSTIC	F		None	SSc/ILD	None
6	MYSTIC	F		MMF/HCQ	SSc/ILD	None
7	MYSTIC	F		HCQ	SSc/ILD	Centromere
8	MYSTIC	F		None	SSc	Centromere
9	MYSTIC	M		None	SSc	RNApol3
10	MYSTIC	M		Pred/IVIG	ARS	Jo-1
11	MYSTIC	M		Pred	ARS	Jo-1
12	MYSTIC	M		None	ARS	Jo-1
13	MYSTIC	F		Pred	ARS	Jo-1
14	BLISS	F		None	SjS/ATD/T1D	SSA, SSB, ANA, RF
15	BLISS	F		HCQ	SjS/CL	SSA, SSB, ANA
16	HIDI	F		None	ATD	Unknown
			56 ± 13			

a BLISS, B Lymphocytes in Sjögren’s syndrome; HIDI, Human Immunology Discovery Initiative; MYSTIC, Myositis and Scleroderma Treatment and Investigative Center.

b Average ± standard deviation.

c HCQ, hydroxychloroquine; MMF, mycophenolate mofetil; Pred, prednisone.

d ATD, autoimmune thyroid disease; CL, cutaneous lupus; ILD, interstitial lung disease; ARS, anti-tRNA synthetase syndrome; SjS, Sjögren’s syndrome; SSc, systemic sclerosis; T1D, type 1 diabetes.

e ANA, anti-nuclear antibodies; RF, rheumatoid factor.

Donors at risk for type 1 diabetes were recruited at Vanderbilt through their participation in Type 1 Diabetes TrialNet Pathway to Prevention (37). Type 1 Diabetes TrialNet participants were first, second, or third-degree relatives of type 1 diabetes probands that were defined as having pre-symptomatic type 1 diabetes based on a history of positivity for two or more islet autoantibodies (IAA, GAD65, ICA, IA2, or ZNT8, defined in **Table 2**, one of which was insulin autoantibody (IAA). Participants were excluded from this cohort if they previously received insulin therapy or were diagnosed with diabetes at the time of blood draw. Participants were excluded for any of the following: fever 101°F in preceding 48hrs of clinic visit, receiving antibiotic therapy at clinic visit, weight less than 8 kg, known pregnancy, anemia at time of study initiation (Hgb <12), comorbid conditions which would render giving blood samples a risk, chronic use of immune suppressive or depleting medications, prior use of immune modulating medications, inability to meet study visit requirements, ongoing use of Accutane, age <1 year, > 60 years at the time of baseline visit. Participant characteristics are outlined in **Table 2**. This study received IRB approval by Vanderbilt and ancillary study approval by Type 1 Diabetes TrialNet. All data were managed using the REDCap software system (38).

**Table 2:** Type 1 Diabetes TrialNet participant demographics.

SubjectID	Gender	Age range (years)^a^	Oral glucose tolerance test results^b^	Autoimmune disease diagnosis^c^	Islet autoantibody positivity at blood draw^d^
17	F		Impaired	Pre-T1D	IAA, IA2, ICA, GAD65, ZNT8
18	F		Impaired	Pre-T1D	IAA, IA2, ICA, GAD65, ZNT8
19	M		Normal	Pre-T1D	IAA, IA2, ICA, GAD65, ZNT8
20	F		Impaired	Pre-T1D	IAA, IA2, ICA, GAD65, ZNT8
21	M		Impaired	Pre-T1D	IAA, GAD65
		13 ± 6			

a Average ± standard deviation.

b Oral glucose tolerance test results are defined as follows based on blood glucose measurements (mg/dL): Normal: Fasting. <110, 1h < 200, 2h < 140, no symptoms; Impaired: Fasting ≥110, <126, 1h ≥ 200, 2h ≥ 140, <200, no symptoms.

<110, 1h < 200, 2h < 140, no symptoms; Impaired: Fasting ≥110, <126, 1h ≥ 200, 2h ≥ 140, <200, no symptoms.

c Pre-T1D diagnosis based on positivity for at least two islet autoantibodies, including insulin autoantibody (IAA).

d The five islet autoantibodies screened by Type 1 Diabetes TrialNet are, GAD65, glutamic acid decarboxylase 65-kilodalton isoform; IAA, insulin autoantibody; IA2, Islet antigen 2; ICA, Islet cell antibodies; ZNT8, Zinc transporter 8.

### Sample Collection and Processing

Peripheral blood mononuclear cells (PBMCs) were obtained by collecting whole blood into mononuclear cell preparation tubes with sodium heparin or sodium citrate (BD) *via* peripheral venipuncture. Cells were washed twice in PBS and red blood cells were lysed using Ack Lysis Buffer (Gibco). Cells were again washed in PBS and counted. Cells were cooled in 10% dimethyl sulfoxide (DMSO) in fetal bovine serum (FBS) at a rate of -1°C/minute until they reached a temperature of -80°C for 24-72 hours. Cells were then stored in liquid nitrogen until the time of analysis. Viability was assessed at time of sample thawing with ∼ 80% average viability.

### ELISA

384-well ELISA plates (Maxisorp) were coated with either 500 ng/ml goat anti-human Ig (Southern Biotech, 2010-01) in 1X PBS overnight at 4°C or 1 μg/mL recombinant human insulin (Sigma I2643) in borate buffered saline overnight at 37°C to assess total antibody or anti-insulin antibody, respectively. The next day, plates were washed 5X with 0.5X PBS and then blocked with 5% chicken serum in 1X PBS plus 0.5% Tween (1X PBS-T) for 1h at room temperature. Block was decanted from plates, blotted dry, and then stimulated PBMC culture supernatants (diluted 1:2 in 1X PBS-T) were added to the plates. Plates were then incubated for 1.5h at room temperature. Bound antibody was detected either by goat anti-human IgG-HRP (Southern Biotech, 2040-05) or goat anti-human IgM-HRP (Southern Biotech, 2020-05) secondary antibodies diluted in 1X PBS-T. Plates were washed 10X with 0.5% PBS. Finally, TMB Ultra ELISA substrate (Thermo Fisher, PI34029) was added, and plates were read at O.D. 370nm using a microplate reader (SpectraMax M3). Wells with fluorescence values above a cutoff of the mean + 3 standard deviations of a blank well were considered positive. Antibody concentrations were calculated using IgG/IgM standards (Sigma, I4506 and I8260, respectively).

### Flow Cytometry Purification of B Cell Subsets and Analysis

Cells were stained in flow cytometry staining buffer (1X PBS containing 5% FBS, 0.02% EDTA, and 0.1% sodium azide) with the following reactive antibodies and reagents: CD19 (SJ25C1), CD21 (Bu32), CD24 (ML5), CD27 (O323), CD38 (HIT2), IgD (IA6-2), IgG (G18-145), IgM (MHM-88) (BioLegend or BD Biosciences). B cell subsets were purified using a FACSAria sorter (BD Biosciences). Data were analyzed using FlowJo software (Tree Star, Inc.). Jo-1-binding B cells were identified by flow cytometry using GST-tagged Jo-1 antigen we previously cloned and recombinantly expressed, followed by detection with anti-GST secondary antibody (Abcam), as previously described (36).

### B Cell Stimulation

Cryopreserved PBMCs were rapidly thawed, washed, and resuspended in ClonaCell-HY Medium A (StemCell). PBMCs or sorted B cell subsets plated as indicated in figure legends in Medium A with 0.833 μg/ml of CpG (ZOEZOEZZZZZOEEZOEZZZT, Invitrogen) + 0.133 μg/ml each of mouse-anti-human kappa and lambda antibodies (Southern Biotech), as well as 0.033x10^6^ cells/mL viable gamma-irradiated NIH3T3 fibroblasts that were genetically engineered to express cell-surface human CD40L (CD154), secreted human B cell activating factor (BAFF), and human IL-21 (unpublished line related to (39, 40), originally provided to Dr. Smith by Dr. Deepta Bhattacharya; Washington University in St. Louis, St. Louis, MO). These stimuli drive B cells to secrete BCR as antibody to enable screening for ASBCs (29). The mixture was then plated into 96-well (300 μL total volume) or 384-well (100 μL total volume) flat bottom plates (Corning). Plates were incubated with 5% CO_2_ at 37°C and screened for antibody production by ELISA after 1 wk, as described above.

### Statistical Analysis

Standard statistical tests utilized for each experiment are indicated in the corresponding figure legends and significance values were calculated using Prism (GraphPad).

## Results

### BCRs Secreted as Antibody Are Captured From Stimulated Mature Naïve and Memory B Cell Subsets

**Figure 1:**
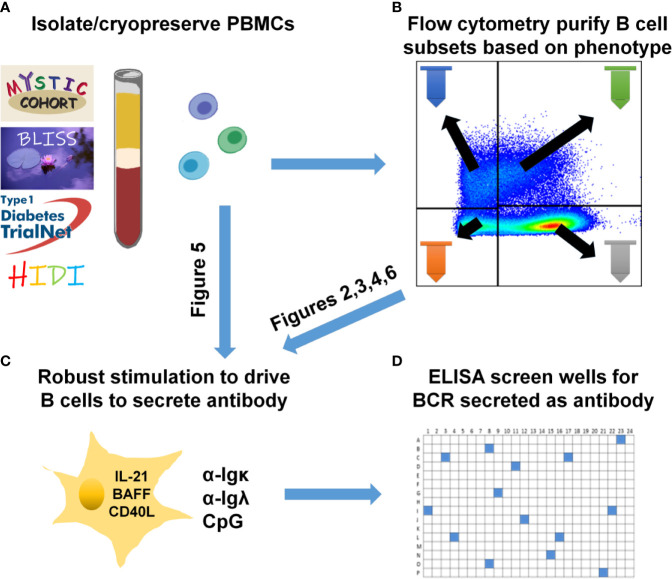
Human B cell stimulation and high-throughput screening methods overview. **(A)** PBMCs were isolated and cryopreserved from the indicated cohorts. **(B)** Specific B cell subsets were flow cytometry-purified. **(C)** As specified for each figure, total PBMCs **(A)** were plated into 96-well plates or purified B cell subsets **(B)** were plated into 384-well plates and stimulated as in Methods to drive B cell proliferation and differentiation into antibody-secreting cells. **(D)** High-throughput ELISA screening of well supernatants identified wells containing ASBCs.

**Figure 2:**
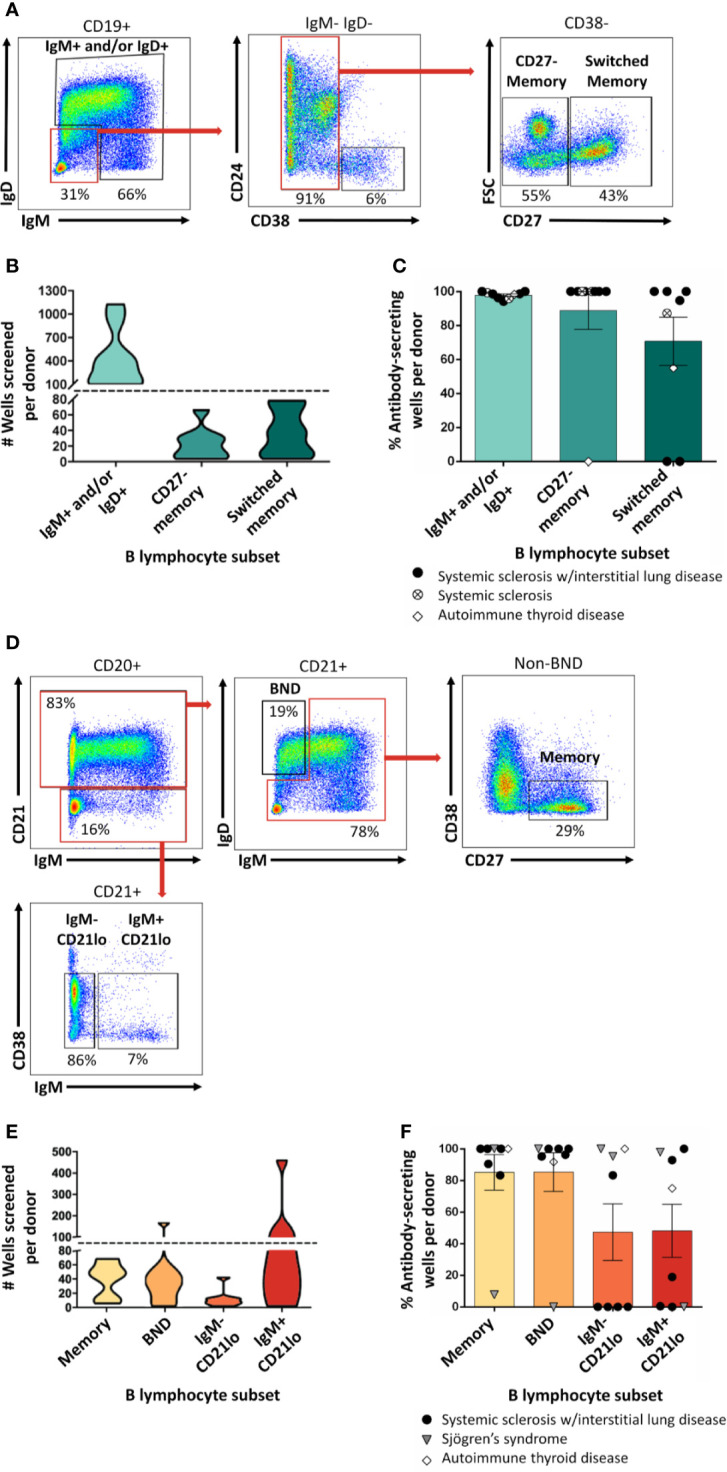
Mature naïve, memory, and autoreactive-prone B cell subsets differentiate into antibody-secreting cells following *in vitro* stimulation. **(A)** Representative plots show flow cytometry identification of the indicated B cell subsets (gated on CD19^+^ live singlet lymphocytes). Subsets were defined as follows: IgM^+^ and/or IgD^+^, CD27^-^ memory (IgD^+^ IgM^+^ CD27^-^ CD38^-^), and CD27^+^ switched memory (IgD^-^ IgM^-^ CD27^+^ CD38^-^). **(B)** Violin plots show the data distribution and density for the number of all wells screened for each B cell subset per donor in **(C), (C)** PBMCs were isolated from systemic sclerosis/interstitial lung disease (black circle, n=6), systemic sclerosis (open circle/X, n=2), or ATD patients (open diamond, n=1) and B cell subsets were identified as in **(A)** and flow cytometry purified. Individual B cell subsets were plated in 384-well plates at ~300 B cells/well and stimulated as in Methods for 1 wk. ELISA was used to measure antibody present in culture supernatants. Wells were scored positively for antibody secretion for ELISA OD_370nm_ > 0.5. The frequency of positive wells was calculated per subset, per donor; individual points represent individual donors. **(D)** Representative plots show flow cytometry identification of the indicated B cell subsets (gated on CD19^+^ live singlet lymphocytes). Subsets were defined as follows: memory (IgM^-^/IgD^-^ or IgM^mid/high^ CD21^+^ CD27^+^ CD38^-^), B_ND_ (IgM^low/neg^/IgD^+^ CD21^+^), IgM^-^ CD21^lo^ (IgM^-^ CD27^+^ CD38^mid/neg^), or IgM^+^ CD21^lo^ (IgM^+^ CD21^lo^ CD38^mid/neg^). **(E)** Violin plots show the data distribution and density for the number of all wells screened for each B cell subset per donor in **(F), (F)** PBMCs were isolated from systemic sclerosis/interstitial lung disease (black circle, n=5), Sjögren’s syndrome (filled triangle, n=2), or ATD patients (open diamond, n=1) and B cell subsets were identified as in **(D)** and flow cytometry purified. Individual B cell subsets were plated in 384-well plates at ~ 300 B cells/well and stimulated as in Methods for 1 wk. Wells were scored positively for antibody secretion for ELISA OD_370nm_ > 0.5. The frequency of positive wells was calculated per subset, per donor; individual points represent individual donors.

ASBCs are not always readily detectable in autoimmune individuals. A robust stimulation method allows capture of IgE ASBCs as rare as 1 in 20 million as hybridomas (29). This method relies on high-throughput ELISA screening for antigen-specific BCRs secreted as antibody following robust stimulation. One caveat with this method is that it does not provide information about the phenotypic subset from which the ASBC was derived. B cell phenotype correlates with dramatic differences in functional capacity; anergic B cells (e.g. B_ND_ cells in humans) are functionally silenced for proliferation and differentiation into antibody-secreting cells, but memory B cells are poised to rapidly contribute to protective immune responses (29). Thus, consideration of which subset(s) ASBCs are expanded in provides important information about how autoreactive B cell clones arise and function in the repertoire to promote autoimmunity. We sought to test whether this stimulation/screening method could be adapted to use flow cytometry-purified B cell subsets from autoimmune disease patient peripheral blood as inputs, as this would enable ASBCs identification coupled with phenotypic information, such as whether the ASBC had a memory phenotype *in vivo*. **Figure 1** provides an overview of the methods applied to each figure. As shown in **Figure 2A**, specific B cell subsets were purified using flow cytometry sorting. The focus of this manuscript is on B cells derived from autoimmune donors; we presented detailed immunophenotyping to compare B cell subset percentages between patients with several of the autoimmune diseases highlighted in this manuscript and healthy controls in separate manuscripts (36, 41). Purified B cell subsets (e.g. memory) were subsequently divided across wells and stimulated as in Methods to drive B cells to differentiate into antibody-secreting cells (**Figure 2B**). ELISA detects the presence of BCR secreted as antibody in CD19^+^ IgM^+^ and/or IgD^+^, class-switched CD27+ memory (CD19^+^ IgM^-^ IgD^-^ CD27^+^), and CD27^-^ memory (CD19^+^ IgM^-^ IgD^-^ CD27^-^) B cell subsets (**Figure 2C**). BCR was also detected as antibody from CD27^+^ memory cells which included IgM^+^ cells (**Figure 2F**). These data show our *in vitro* stimulation protocol can be used to screen BCRs expressed by non-class-switched and memory B cell subsets.

### Autoreactive-Prone B_ND_ (Anergic) and CD21^low^ B Cells Are Stimulated to Secrete BCRs as Antibody

We previously identified ASBCs using fluorescently-labeled autoantigen (36). This detection method could however miss B cells that downregulate surface BCR because of chronic antigen stimulation and/or immune tolerance mechanisms such as anergy. Anergy is an immune tolerance mechanism by which autoreactive B cells are functionally silenced but retained in the repertoire; they can be phenotypically identified as B_ND_ cells (IgM^lo/-^ IgD^+^ CD27^-^) (32). CD21^lo^ B cells are an autoreactive-prone subset that is increased in the peripheral blood of patients with several autoimmune diseases (31, 33, 42). CD21^lo^ B cells have been reported to be anergic, yet also to express highly mutated BCRs that are consistent with germinal center origin (31, 33, 43). Given the potential for BND and CD21^lo^ B cell subsets to serve as autoreactive B cell reservoirs, we flow cytometry purified these subsets as shown in **Figure 2D** and screened wells to determine (**Figure 2E**) whether our stimulation protocol could drive B cell subsets to differentiate and secrete their BCRs as antibody (44). B_ND_ and CD21^lo^ BCRs were detected as antibody following stimulation (**Figure 2F**), demonstrating that BCRs from these subsets can be evaluated using this protocol.

### Plasmablast Secretion of Antibody Is Not Sustained Following *In Vitro* BCR/CD40/BAFF/Cpg/IL-21 Stimulation

**Figure 3:**
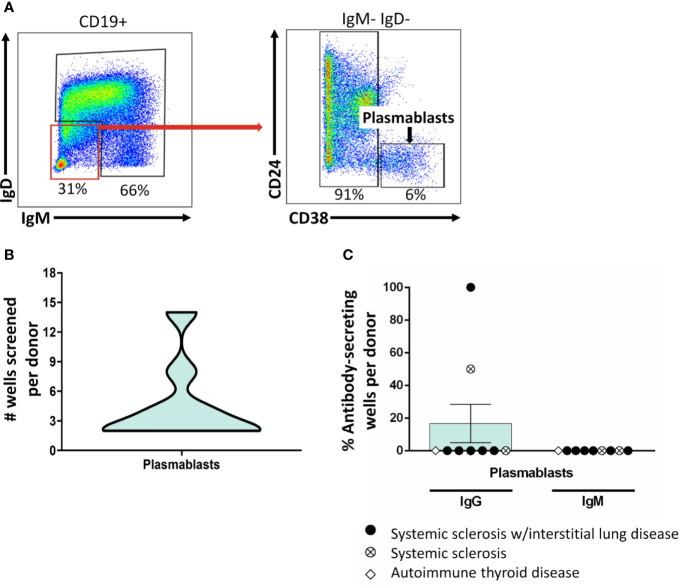
Class-switched plasmablasts do not sustain antibody secretion following BCR/CD40/BAFF/TLR9/IL-21 stimulation. **(A)** PBMCs were isolated from autoimmune disease patients. Representative plots show flow cytometry identification of plasmablasts (CD19^+^ IgD^-^ IgM^-^ CD24^-^ CD38^-^ live singlets). **(B)** B cell subsets were identified as in **(A)** and flow cytometry purified. Plasmablasts were plated in 384-well plates at ~300 plasmablasts/well and stimulated as in Methods for 1 wk. The data distribution and density of the number of plasmablast wells that were screened per donor is expressed as a violin plot. **(C)** Culture supernatants from plasmablasts plated as in **(B)** were screened for antibody production by ELISA. Wells were scored positively for antibody secretion for ELISA OD_370nm_ > 0.5. The frequency of positive wells was calculated per subset, per donor; individual points represent individual donors. Results from systemic sclerosis/interstitial lung disease (black circle, n=6), systemic sclerosis (open circle/X, n=2), or ATD patients (open diamond, n=1) are shown.

B cells do not secrete antibody; rather, if they receive the right signals, they differentiate into antibody-secreting cells. Whereas plasmablasts are short-lived antibody-secreting cells that can be found in peripheral blood, plasma cells are long-lived antibody-secreting cells that can be found in the bone marrow and other tissues. Plasmablasts are enriched for ASBCs, thus we wanted to test whether this approach captures plasmablast BCRs. Flow cytometry purification identified plasmablasts as in **Figure 3A**, which were stimulated *in vitro* as in Methods (**Figure 3B**). Antibody was not detected by ELISA for plasmablast isolated from the majority of donors following this stimulation protocol (**Figure 3C**), thus other approaches are required to assay antigen-specific plasmablasts.

### Limited Class-Switching Is Observed for IgM^+^/IgD^+^ B Cells Following *In Vitro* Stimulation

**Figure 4:**
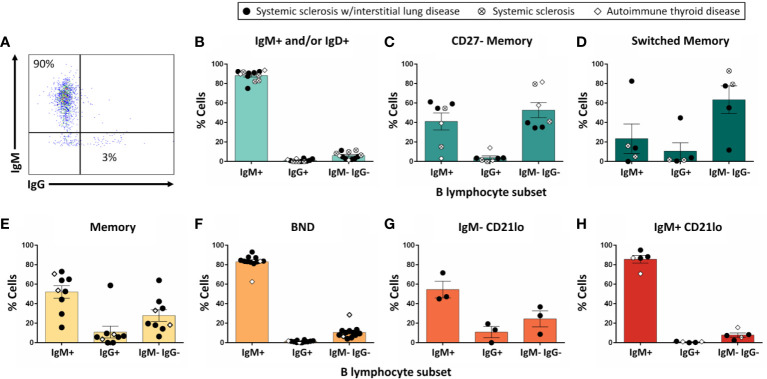
The majority of IgM^+^ and/or IgD+ B cells do not undergo class-switch to IgG following *in vitro* stimulation. PBMCs were isolated from systemic sclerosis/interstitial lung disease (black circle, n=6 donors; n=35 wells), systemic sclerosis (open circle/X, n=2; n=8 wells), or ATD patients (open diamond, n=1; n=11 wells). Flow cytometry-purified B cell subsets (identified as in **Figure 1**) were plated in 384-well plates at ∼300 cells/well and stimulated as in Methods for 1 wk. Flowcytometry was used to measure the frequency of IgM^+^, IgG^+^, and IgM^-^ IgG^-^ cells among CD19^+^ live singlet lymphocytes **(A)**. Flow phenotyping inclusion criteria for this analysis was > 95 lymphocyte events and > 35 CD19^+^ events. Results for **(B)** IgM^+^ and/or IgD^+^, **(C)** CD27^-^ memory, **(D)** CD27^+^ switched memory, **(E)** memory, **(F)** BND, **(G)** IgM^-^ CD21lo, and **(H)** IgM^+^ CD21lo B cell subsets are shown.

Non-class-switched B cell subsets were stimulated for 6d as in Methods to test whether they undergo class-switching to IgG (**Figure 4A**). Flow cytometry analysis revealed limited class-switching away from the IgM isotype for IgM^+^ and/or IgD^+^, B_ND_, or IgM^+^ CD21^lo^ B cells following stimulation (**Figures 4B, F, H**). Conversely, the majority of switched memory B cells were identified as IgG^+^ or IgM^-^ IgG^-^ at the end of culture (**Figure 4D**), potentially indicating IgA class switch, which was not measured in this assay. The memory B cell subset, which included both IgM^+^ and IgM^-^ B cells as input, contained both IgM^+^ and switched B cells following stimulation (**Figure 4E**), suggesting both IgM^+^ and class-switched CD27^+^ B cells survive in this culture. IgG^+^ and IgM^-^ IgG^-^ B cells were observed among cultured CD27^-^ memory and IgM^-^ CD21^lo^ B cells as expected (**Figures 4C, G**), however the relatively high proportion of IgM^-^ CD21^lo^ B cells that expressed IgM at the end of culture suggests class-switched B cells in these subsets may not respond as strongly as IgM^+^ CD21^lo^ B cells to this stimulation.

### Insulin-Binding ASBCs Are Detected Among Memory B Cell Subsets From Donors at High Risk for Type 1 Diabetes

**Figure 5:**
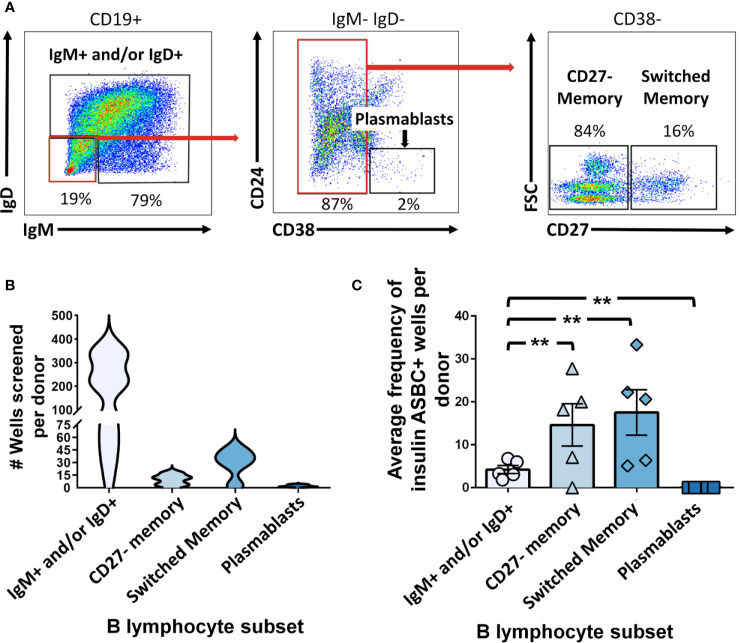
Insulin-binding ASBCs are identified within specific B cell subsets. **(A)** Representative plots show flow cytometry identification of the indicated B cell subsets (gated on CD19^+^ live singlet lymphocytes). Subsets were defined as follows: IgM^+^ and/or IgD^+^, CD27^-^ memory (IgD^+^ IgM^+^ CD27^-^ CD38^-^), and CD27^+^ switched memory (IgD^-^ IgM^-^ CD27^+^ CD38^-^). **(B)** Data distribution and density of wells screened per donor are expressed as a violin plot per subset. **(C)** PBMCs were isolated from n=5 participants at high risk for type 1 diabetes [positive for ≥ two islet autoantibodies, one of which was insulin autoantibody (IAA)]; flow cytometry-purified B cell subsets (identified as in **Figure 1**) were plated in 384-well plates at ~300 cells/well and stimulated as in Methods for 1 wk. ELISA was used to measure anti-insulin antibody in culture supernatants to identify wells containing insulin-binding ASBCs (positive wells defined as O.D._370nm_ ≥ 0.85 which was one standard deviation above the mean and which displayed reduced insulin antibody binding when parallel supernatants were incubated with 100-fold excess soluble insulin competitor). Errors bars shown are SEM per subset. Each data point represents the average % insulin-binding ASBCs identified per donor for each B cell subset. **p < 0.01, Mann-Whitney U test.

ASBCs are not always readily detectable in autoimmune individuals. For example, *ex vivo* detection of anti-insulin B cells is challenging in both humans and mice at high risk for type 1 diabetes, despite being seropositive for insulin autoantibody (22, 32). We sought to couple phenotypic information with our ASBC screening pipeline to provide methods to better understand how ASBC functional capacity changes with respect to autoimmune disease progression, or in response to immune therapy using small volumes (2-10mL) of peripheral blood. Whereas CD27^+^ memory B cells arise through germinal center reactions, CD27^-^ IgD^-^ memory B cells (increased in the autoimmune disease, SLE) do not (45, 46). Insulin is a major autoantigen in type 1 diabetes and insulin autoantibodies are one of five islet autoantibodies used by Type 1 Diabetes TrialNet to identify individuals at high risk for type 1 diabetes (3). Donors at risk for type 1 diabetes were identified as in Methods based on donor positivity for insulin autoantibody (IAA) plus at least one other islet autoantibody and PBMCs were flow cytometry sorted to identify IgM^+^ and/or IgD^+^, IgD^-^ CD27^-^ memory, or CD27^+^ memory B cells and stimulated as in Methods (**Figure 5A**). Wells that contained insulin-binding B cells were identified by ELISA using a secondary antibody confirmed to bind both IgM and IgG to ensure fair comparison across subsets regardless of isotype (**Figure 5B**). An increased frequency of CD27^-^ and CD27^+^ memory subset wells contained insulin-binding ASBCs relative to the IgM^+^ and/or IgD^+^ subset, suggesting insulin-binding B cells are enriched among memory B cells (**Figure 5C**). Thus, these approaches can be used to detect expanded ASBCs within specific B cell subsets.

### Jo-1-Binding ASBCs Expand Following *In Vitro* Stimulation

**Figure 6:**
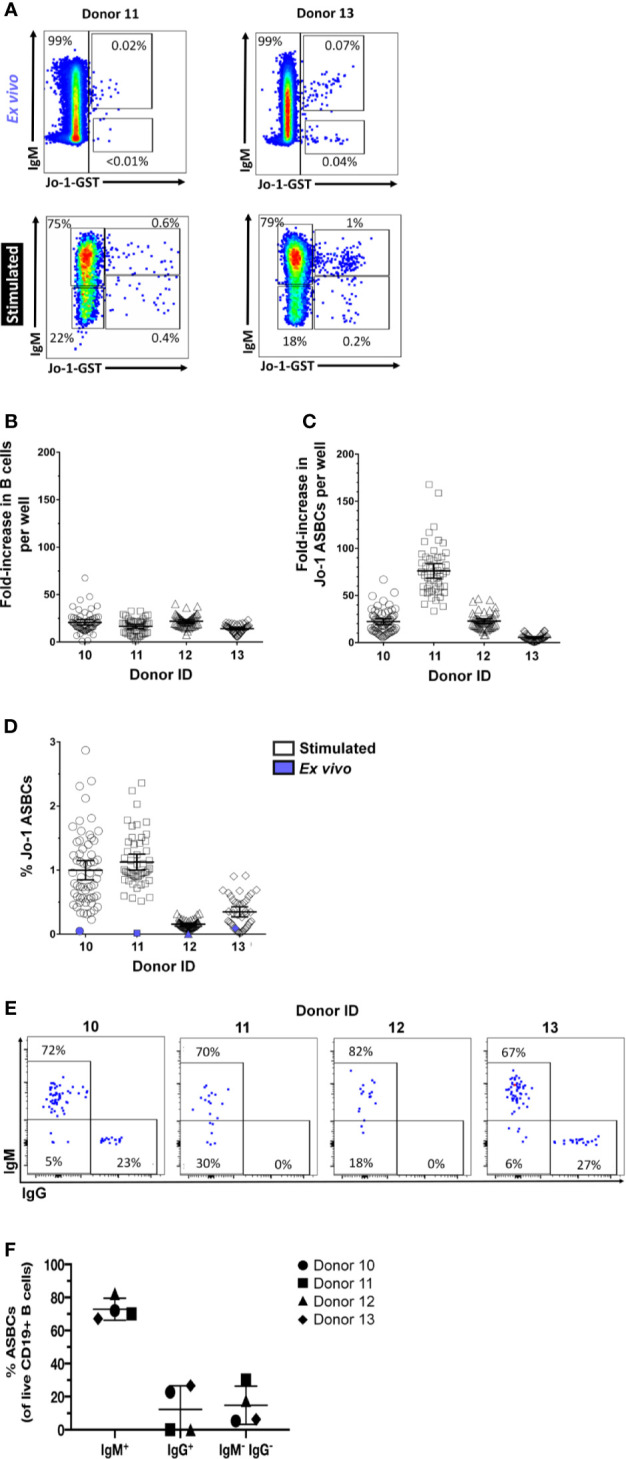
Autoreactive Jo-1-binding ASBCs expand in response to *in vitro* stimulation. PBMCs were isolated from n = 4 Jo-1 anti-histidyl tRNA synthetase syndrome patients, and **(A)** representative plots show flow cytometry identification of total B cells as well as Jo-1-binding ASBCs *ex vivo* and following stimulation *in vitro* for 1 wk as in Methods (gated on CD19^+^ live singlet lymphocytes). 3.3x10^4^ PBMCs were stimulated per well in 96 well plates as in Methods. An average input total B cell and Jo-1-binding B cell frequency per well was calculated based on *ex vivo* flow phenotyping data. After 1 wk of stimulation, individual wells were harvested and flow cytometry was used to determine output total B cell and Jo-1-binding ASBC frequency per well. Output frequency/input frequency was used to calculate estimated fold expansion for **(B)** total B cells or **(C)** Jo-1-binding ASBCs in each well. Individual wells are plotted. **(D)** The frequency of Jo-1-binding ASBCs *ex vivo* (purple symbol) or following *in vitro* stimulation (open symbols) is shown for each donor; individual wells are plotted. **(E)** Representative plots show flow cytometry identification of Jo-1-binding ASBCs by IgM and IgG isotypes (gated on Jo-1-GST^+^ CD19^+^ live singlet lymphocytes). **(F)** The isotype frequency of Jo-1-binding ASBCs following *in vitro* stimulation is shown for each donor; individual donors are plotted.

Flow cytometry detection of ASBCs can be challenging due to low frequency of target cells. However, we recently identified a readily detectable population of Jo-1-binding B cells in the peripheral blood of Jo-1 autoantibody-positive anti-histidyl tRNA synthetase syndrome patients using flow cytometry staining with labeled autoantigen (36). This created an opportunity to directly examine expansion of ASBCs following our *in vitro* stimulation protocol. The frequency of Jo-1-binding B cells was measured for n=10 Jo-1+ anti-histidyl tRNA synthetase syndrome donors; PBMCs from n=4 of these donors were stimulated for 1 wk as in Methods and cells from individual wells were flow cytometry phenotyped (**Figures 6A, E**) to determine the frequency of Jo-1-binding B cells (ASBCs), as well as total B cells following stimulation. B cells underwent an average 18-fold expansion (**Figure 6B**), whereas Jo-1-binding ASBCs expanded 32-fold on average (**Figure 6C**) following *in vitro* stimulation. These data confirm that Jo-1-binding ASBCs expand at least as well as total B cells *via* this stimulation protocol. PBMCs are divided across 384-well plates, enabling bias for particular wells towards a given antigen specificity. This is illustrated in **Figure 6D**, which shows the relative frequency of ASBCs is increased following *in vitro* stimulation as compared to the *ex vivo* frequency of ASBCs. Additionally, we showed a heterogeneity across IgM and IgG isotypes within Jo-1-binding ASBCs across different donors (**Figure 6F**). A small percentage of Jo-1-binding ASBCs were isotypes other than IgM and IgG (5-30%; IgM^-^, IgG^-^).

This approach could thus be applied to expand the original number of ASBCs to support downstream assays for which ASBC numbers are a limiting factor.

## Discussion

Specific B cell subsets function differently to coordinate protective or autoimmune responses. Hybridoma technology advancements enable capture of ASBCs as rare as 1 in 20 million cells (29), but this approach does not capture B cell subset phenotypic data. We present data to show that flow cytometry-purified B cell subsets can be stimulated using this published protocol to additionally discern the phenotypic subset of ASBCs. Knowledge about which subset (e.g. memory) ASBCs derive from and how this might change with disease progression could enhance efforts to track ASBCs as sensitive disease biomarkers to predict increasing disease severity or flares, or alternatively as barometers for immunomodulatory therapy success.

Different autoimmune diseases may arise through distinct immune tolerance checkpoint breaches. B cell differentiation into an antibody-secreting cell may be governed differently for different ASBC specificities and across different diseases; for example, whereas Ro60 autoantibodies are typically sustained and Jo-1 autoantibody levels track with IIM disease severity, insulin autoantibodies are frequently transient (25, 47–49). To encompass this potential heterogeneity, B cell subset responses were surveyed from patients with several autoimmune diseases. We find that most of the B cell subsets tested secrete BCR as antibody using this stimulation protocol. Mature naïve, B_ND_, CD27^+^ memory, IgM^-^ IgD^-^ CD27^+^ memory, IgM^-^ IgD^-^ CD27^-^ memory B cell subsets isolated from at least 70% of patients responded to this stimulation protocol by secreting antibody. This study was underpowered to uncover specific functional differences present among B cells isolated from patients/participants with different autoimmune diseases. Additional studies will be required to uncover autoimmune disease-specific differences in B cell subset responses to this stimulation protocol.

Responses were more variable among IgM^-^ CD21^lo^ and IgM^+^ CD21^lo^ B cell subsets; for each subset, roughly 50% of patients showed a response. Of note, an abnormal CD19^+^ IgM^-^ IgD^-^ CD21^lo^ subset has been identified in systemic sclerosis and anti-histidyl tRNA synthetase syndrome, which expresses many innate immune cell markers (unpublished observations). If these cells are not of the B lineage, that would explain their inability to secrete antibody. Alternatively, if CD21^lo^ B cells have already upregulated a plasmablast/plasma cell transcriptional program, as has been suggested in the setting of infection (43), they may respond less well to stimulation, as plasmablast responses were largely absent with this stimulation protocol. Given the known functional difference identified among the broad category of CD21^lo^ B cells (31, 33, 43), our data highlight the need to test this method in specific autoimmune diseases in which aberrant CD21^lo^ B cell populations have been identified.

The limited antibody secretion detected from plasmablasts following this *in vitro* stimulation points to a need to apply other approaches, such as single-cell BCR sequencing, to examine the antigen-specific plasmablast repertoire. Alternatively, this stimulation protocol could be modified to include factors essential for plasmablast/plasma cell survival and differentiation, such as IL-6, IL-10, IFN-α and APRIL (50, 51). Such modifications would need to be separately tested for their impact on other B cell subsets. Innate stimulation, such as through TLR7 and TLR9, is known to drive activation of autoreactive B cells in systemic lupus erythematosus (52, 53). Studies of murine anti-insulin B cells showed that LPS (TLR4), but not ssDNA (TLR7) or CpG (TLR9) stimulation, overrode immune tolerance checkpoints to drive anti-insulin B cell proliferation (54). The innate stimuli that can drive immune tolerance breach by human anti-insulin B cells have not been investigated. The data presented here confirm insulin and Jo-1-binding B cells are captured using this method; we also identify Ro-52 and Ro-60-binding B cells using this method (unpublished observations).

Purified B cells that were IgM^+^ or IgD^+^ underwent limited class-switching away from the IgM isotype following *in vitro* stimulation, suggesting isotype is largely preserved for non-class-switched cells. Co-expression of multiple IgH transcripts has been noted in memory B cells and is enhanced in patients with Sjögren’s syndrome (55). Thus, it is possible that the limited IgG class switching among memory-phenotype B cells may reflect transcriptional programming that occurred *in vivo*, rather than in response to stimulation *in vitro*. IgG^+^ B cells were detected among stimulated IgM^-^/IgD^-^ B cell subsets, as expected, but many cells surveyed were negative for both IgM and IgG surface expression. IgM^-^/IgG^-^ cells may be of the IgA isotype, which was not examined in this study. B cells terminally differentiated to the IgE isotype are captured by this stimulation protocol (29), but future studies are warranted to confirm whether IgA^+^ B cells can be assessed using this method.

The ease with which ASBCs can be studied depends on repertoire frequency, as well as the sensitivity of the technology employed. Jo-1-binding ASBCs were detected in 50% of Jo1+ anti-histidyl tRNA synthetase syndrome patients surveyed (36), whereas magnetic sorting enrichment of insulin-binding ASBCs was used to overcome their low frequencies in at-risk/type 1 diabetic donors (32). We took advantage of the relatively high frequency of Jo-1-binding B cells *ex vivo* to measure autoreactive ASBC expansion in this culture system. Jo-1-binding ASBCs expanded 32-fold on average, suggesting this approach might be coupled with other downstream assays in which expansion of ASBCs is necessary.

**Figure 7:**
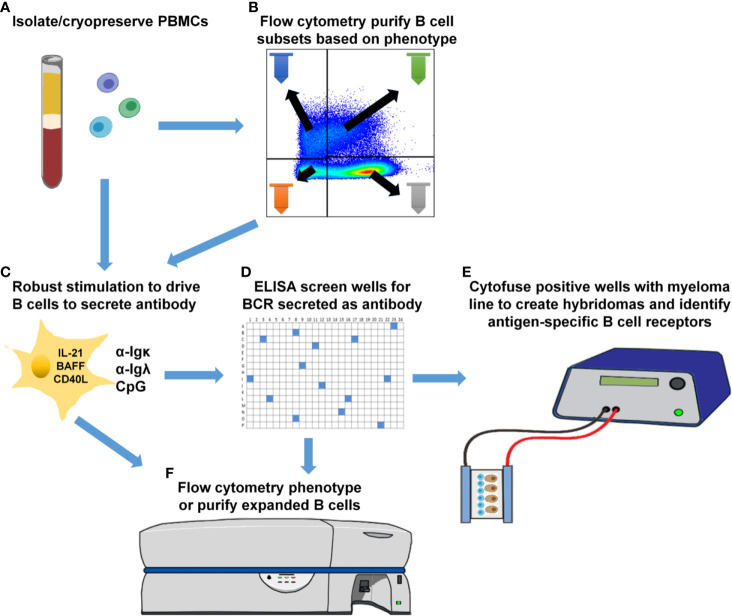
Downstream applications for methods to phenotypically-define, expand, and identify ASBCs. **(A)** Isolate and cryopreserve PBMCs. **(B)** Optionally purify specific B cell subsets using flow cytometry cell sorting. **(C)** Plate total PBMCs (panel **A**) or purified B cell subsets (panel **B**) into 384-well plates and stimulate as in Methods to drive B cell proliferation and differentiation into antibody-secreting cells. **(D)** High-throughput ELISA screening of well supernatants to identify wells containing ASBCs. **(E)** Cytofuse candidate wells to produce human hybridoma lines or **(F)** enrich for ASBCs prior to flow cytometry phenotyping.

In this study, we identify insulin-binding ASBCs among CD27^+^ and CD27^-^ memory subsets in donors at high risk for type 1 diabetes, whereas relatively fewer wells derived from IgM^+^ and/or IgD^+^ (presumably mature naïve) B cells from the same donors scored positive for the presence of anti-insulin ASBCs. Polyreactive B cells that bind several antigens, including insulin, have been identified in autoimmune donors among recent bone marrow emigrants and mature naïve B cells (56–58). These data suggest that if polyreactive B cells were responsible for the anti-insulin ASBCs identified, they should be skewed away from, not towards class-switched memory subsets. One limitation of this approach is that multiple B cell clones (and thus antibody specificities) are present in each well; this ELISA-based approach limits accurate assessment of polyreactivity of ASBCs, which are not assayed separately from non-ASBCs present in the same well. Parallel ELISA screening using excess unlabeled antigen competitor is thus the most reliable method for determining antigen specificity, which we applied in this study to identify wells containing anti-insulin ASBCs. Individual B cell receptor binding attributes must be assayed directly to confirm autoantigen specificity; as such, efforts are presently underway in our laboratory to interrogate B cell receptors isolated from ASBCs present in specific B cell subsets that were immortalized as hybridomas. We demonstrate this stimulation protocol can expand ASBCs, thus it may also be amenable to combination with downstream single-cell profiling that can combine BCR repertoire (BCRseq), transcriptional profiling (RNAseq), phenotypic profiling (CITEseq), and/or antigen-reactivity profiling (LIBRAseq) (59, 60). This stimulation protocol produces B cells with sufficient viability that they can be fused to myelomas as hybridoma lines (29). We presume it will also be amenable to combination with these single-cell techniques, but this will require confirmation in future studies. **Figure 7** shows examples of downstream applications that could be coupled to B cell subset purification, stimulation, and high-throughput screening presented in this report, which include the development of human hybridoma cell lines to interrogate B cell receptor sequence and function.

## Data Availability Statement

The original contributions presented in the study are included in the article/supplementary material. Further inquiries can be directed to the corresponding author.

## Ethics Statement

The studies involving human participants were reviewed and approved by Vanderbilt Institutional Review Board, Vanderbilt University Medical Center. The patients/participants provided their written informed consent to participate in this study.

## Author Contributions

BJ, JJ, AS, AC, and RB designed and performed experiments and analyzed resulting data. SS designed experiments and provided critical feedback. DM, LC, and EW recruited donors for these studies. BJ and RB wrote the manuscript. All authors contributed to the article and approved the submitted version.

## Funding

National Institutes of Health Grants NIH T32AR059039, and K12HD043483, Vanderbilt Institute for Clinical and Translational Research Pilot and Feasibility Grant VR53141 (supported by Vanderbilt NIH/CTSA UL1 RR024975), Vanderbilt NIH/CTSA UL1TR000445, Juvenile Diabetes Research Foundation Strategic Research Agreement 3-SRA-2019-790-S-B, Porter Family Fund for Autoimmunity Research, the Vanderbilt Medical Research Student Research Training Program in Diabetes and Obesity [supported by the Vanderbilt Short Term Research Training Program for Medical Students (NIH grant DK007383), Vanderbilt Research Training in Diabetes and Endocrinology (NIH grant T32DK007061), and the Vanderbilt Diabetes Research and Training Center (NIH grant DK20593)], and the Vanderbilt Human Immunology Discovery Initiative supported this work, along with the Vanderbilt Medical Center Flow Cytometry Shared Resource [supported by Vanderbilt Ingram Cancer Center (P30 CA68485) and the Vanderbilt Digestive Disease Research Center (DK058404)].

## Conflict of Interest

The authors declare that the research was conducted in the absence of any commercial or financial relationships that could be construed as a potential conflict of interest.

## Abbreviations

ASBCs, Antigen-specific B cells; BCR, B cell receptor; IAA, insulin autoantibody; PBMCs, peripheral blood mononuclear cells.
